# Audiological profile and cochlear functionality in Williams syndrome

**DOI:** 10.1590/2317-1782/20212021041

**Published:** 2022-01-12

**Authors:** Liliane Aparecida Fagundes Silva, Rachel Sayuri Honjo Kawahira, Chong Ae Kim, Carla Gentile Matas

**Affiliations:** 1 Departamento de Fisioterapia, Fonoaudiologia e Terapia Ocupacional, Faculdade de Medicina – FM, Universidade de São Paulo – USP – São Paulo (SP), Brasil.; 2 Unidade de Genética, Faculdade de Medicina – FM, Instituto da Criança, Hospital das Clinicas – HC, Universidade de São Paulo – USP – São Paulo (SP), Brasil.

**Keywords:** Williams Syndrome, Hearing loss, Cochlea, Audiology, Audiometry Pure-tone

## Abstract

**Purpose:**

to evaluate cochlear functionality in Williams syndrome (WS) individuals.

**Methods:**

a study with 39 individuals, being 22 with WS aged between 7 and 17 years, 15 male and 7 female, and 17 individuals with typical development and normal hearing. All individuals were evaluated using pure tone audiometry, acoustic immittance measurements, and Transient Evoked Otoacoustic Emissions (TEOAE). The audiological profile in individuals with WS was analyzed, and TEOAE responses were compared between WS individuals without hearing loss and typical developmental individuals.

**Results:**

The hearing loss was observed in 50% of patients, being 78.95% sensorineural and 21.05% mixed. This hearing loss was predominantly mild to moderate, affecting mainly frequencies above 3 kHz. As for TEOAE, there was a higher incidence of absence and lower amplitude responses in individuals with WS.

**Conclusion:**

WS individuals have hair cell dysfunction, mainly in the basal region of the cochlea. Thus, TEOAE analysis is an important clinical resource to be considered in the routine audiological evaluation.

## INTRODUCTION

Williams syndrome (WS) is a rare genetic disorder resulting from a microdeletion in the region of the long arm of chromosome 7 (7q11.23), containing approximately 28 genes^(1,2)^. It is estimated to occur in 1:7.500 live births and has an equal prevalence in male and female individuals^(1)^. The WS phenotype is characterized by multiple physical, neurological, and systemic abnormalities^(2)^.

In the scope of the audiological phenotype, especially at high frequencies, above 3 kHz, sensorineural hearing loss has been pointed out in several studies addressing the WS^(3-5)^, an involvement that starts in the late childhood period and early adult life and tends to be progressive^(3,5-7)^.

Seeking to understand such a scenario, some studies have associated the influence of some genes often deleted in individuals with WS that can contribute to their hearing loss, including the *Elastin* (*ELN*)^(1)^ gene, *General Transcription Factor* (*GTF2*I) gene, and *Lim Domain Kinase 1* (*LIMK1*) gene^(8)^. Many studies have observed the expression of these genes in the tissue of the auditory system, damaging blood supply, balance of ion gradient, and functionality of cochlear structures^(3,9,10)^. Therefore, especially regarding cochlear functionality, the audiological assessment of WS is highly important for this population.

A way of measuring the functionality of outer hair cells of the cochlea is by capturing Otoacoustic Emissions (OAE), which are responses generated by the energy release in these cells and can occur spontaneously or evoked by an acoustic stimulus. The most commonly used stimuli include the transient-evoked otoacoustic emission (TEOAE), consisting of a brief stimulation through clicks or tone burst, and the Distortion Product (DPOAEs), in which two pure tones of different frequencies are emitted simultaneously generating a reflected response that is a product of the distortion resulting from the combination of the two tones^(11)^.

Some OAE studies have demonstrated a cochlear fragility since even individuals with normal auditory thresholds can present cochlear alterations accompanied by subclinical symptoms^(4,5,12,13)^. However, hearing-related studies in patients with WS are still very recent and scarce. Although different hypotheses have been raised since the early studies aiming to find responses that justify the behavioral phenotype of these individuals, little is known on the origin of most auditory characteristics of this population.

Therefore, our goal is to characterize the audiological profile and assess the cochlear functionality of individuals with WS.

## METHODS

This is a transversal clinical study approved by the Ethics Committee of the research institution, protocol number 15.825, carried out in individuals with WS (Study Group – SG) attended at the Genetics Unit of the Children's Institute of the Clinical Hospital of the Medicine School, University of São Paulo.

The following inclusion criteria were applied for the individuals in the SG: to present confirmed WS diagnosis through the Multiplex Ligation-dependent Probe Amplification (MLPA) and belong to the age group between seven and 17 years old. The exclusion criteria were excess wax in the external acoustic meatus and failure to comply with all procedures.

We performed a clinical analysis of the records that resulted in the selection of 34 patients to participate in the study. Four of these patients could not be reached, six did not agree to participate, one had excess wax in the external acoustic meatus and did not return for evaluation after removal, and one did not allow some of the procedures.

Thus, 22 patients were excluded from the study: seven females and 15 males with chronological age between seven and 17 years (12.36 ± 3.02) and mental age between three and 14 years (6.52 ± 2.28). The Intelligence Quotient (IQ) assessment based on the Wechsler Abbreviated Intelligence (WASI) revealed results between 37 and 98 (54.05 ± 13.48).

For comparison purposes, a Control Group (CG) was composed of a convenience sample containing 17 individuals with typical development and normal hearing – seven female and 10 male – of chronological age between seven and 17 years (11.88 ± 3.12). These individuals did not present any complaints of delayed cognitive neuropsychomotor, speech, or school performance development, which were verified through anamnesis with the respective parents or caregivers.

To emphasize the presence of hearing loss or middle ear involvement in the patients in the CG, we considered the following inclusion criterion: the presence of tympanometric curve types A, Ad or Ar and ipsi-and contralateral acoustic reflexes at the frequencies of 0.5, 1, 2, and 4 kHz^(14)^; auditory thresholds obtained through pure tone audiometry below 20 dB NA at all tested frequencies (0.25, 0.5, 1, 2, 3, 4, 6, and 8 kHz)^(15)^; Speech Recognition Threshold up to 10 dB HL above the three-tone average, and Speech Recognition Percentage Index containing at least 88% of hits^(15)^.

Before starting the procedures, the parents read and signed an Informed Consent Form, and the patients signed a Term of Consent. Next, the parents answered an audiological anamnesis to provide the information from the patients’ records regarding factors that could interfere with the assessment. Additionally, a meatuscopy was performed to verify a possible obstruction in the external acoustic meatus.

We performed acoustic immittance measures to verify possible middle ear involvement on an immittance meter by Interacustic, model AT235, equipped with a 226 Hz probe. According to the peak admittance measure, the tympanometric curve was characterized following Jerger’s criteria^(14)^. The acoustic reflexes were tested manually until the intensity of 110 dB HL and retested in cases of reflex absence to confirm the results.

Subsequently, we conducted pure tone and speech audiometry using a clinical audiometer by Grason-Stadler, model GSI 61, with the patient inside the acoustic booth, according to regulation ANSI S3.1-1991 for the amount of environmental noise. Pure tone air-conduction audiometry thresholds were measured at the conventional frequencies (0.25 to 8 kHz) considering values up to 20 dB HL^(15)^ as normal auditory thresholds. Upon thresholds above 20 dB HL at any frequency between 0.5 and 4 kHz, bone-conduction audiometry was performed to determine the type of hearing loss^(15)^. To establish the hearing loss degree, we considered each frequency individually as follows: from 25 to 40 dB HL – mild; 45 to 70 dB HL – moderate; 75 to 90 dB HL – severe; and above 95 dB HL – deep. The Speech Recognition Threshold (SRT) was applied in the speech audiometry aiming at a lower intensity at which the individual had at least 50% of hits for trisyllable or polysyllable spoken words, in addition to the Speech Recognition Percent Index (SRPI), with a list of 25 monosyllables to be spoken at the intensity of 30 dB HL above the SRT, considering hits above 88% as normal results^(15)^.

The Transient Evoked Otoacoustic Emissions (TEOAE) were measured only in patients who presented normal auditory thresholds using the equipment Smart EP USB Jr by Intelligent Hearing Systems (IHS 5020) (Miami-Florida), equipped with a 10D phone placed on the patient’s acoustic meatus with latex rubber. The collection parameters encompassed the acquisition of 1024 click stimuli with a non-linear 75-microsecond duration at a rate of 19.30 stimuli per second, a gain of 4000 at the intensity of 80 dB SPL. The analysis window was kept open at 30 ms analyzing at the frequencies of 1, 1.5, 2, 3, and 4 kHz. To determine the presence of TEOAE, we established probe stability of ≥70%, response reproductivity of ≥50%, and applied the criterion of signal-noise relation (S/R) higher than 3 dB SPL for the frequencies of 1 and 1.5 kHz and higher than 6 dB SPL for the remaining frequencies. As for the response, we considered its presence in at least three frequency bands. In patients without TEOAE indication, the probe was repositioned and another measurement was performed considering the best response achieved.

The data collected were tabulated and descriptive and inferential statistical analyses were performed through parametric tests since the sample followed a normal distribution. An unpaired T-test was applied to compare the control and study groups, while a Pearson's chi-square test (χ^2^) verified the association between two variable categories, such as groups and presence/absence of response.

The statistical analysis of the data adopted a significance level of ≤ 0.05 (5%). Values regarded as statistically significant are marked with an asterisk (*).

## RESULTS

Although only two families have reported being aware of the patient’s hearing loss, our study found 11 patients (19 ears) who presented hearing loss (50% of the sample) (Table 1). Among the ears with hearing loss, we observed a higher prevalence of sensorineural hearing loss (15/19 ears = 78.95%), followed by mixed hearing loss (4/19 ears = 21.05%).

**Table 1:** Summary of results obtained from the tympanometric curve and pure tone audiometry of patients with hearing loss

**Patient**	**Right Ear**				**Left Ear**			
	**Type of Tympanometric curve**	**Hearing loss**			**Tympanometric curve**	**Hearing loss**		
		**Type**	**Degree**	**Frequencies (kHz)**		**Type**	**Degree**	**Frequencies (kHz)**
1	A	SNHL	Mild	6 and 8	A	SNHL	Mild	6 and 8
2	A	SNHL	Mild to moderate	6 and 8	A	SNHL	Mild	6 and 8
3	A	Normal thresholds			A	SNHL	Mild	3 and 6
4	C	SNHL	Mild	6 and 8	C	SNHL	Mild	6 and 8
5	A	SNHL	Mild	0.25 and 0.5	A	Normal thresholds		
6	A	SNHL	Mild	4	A	Normal thresholds		
7	Ad	SNHL	Mild	0.25 and 6	Ad	SNHL	Mild	0.25
8	A	SNHL	Moderate	3 to 8	A	MHL	Mild to severe*	All
9	B	MHL	Moderate to severe*	All	A	SNHL	Mild to moderate	4 to 8
10	Ad	SNHL	Mild	6 and 8	A	SNHL	Mild	1, 2, 3, 6, and 8
11	B	MHL	Mild to moderate*	All	B	MHL	Mild to moderate^*^	All

* degree of hearing loss considering the air-conduction thresholds

**Caption:** kHz- Kilo Hertz; SNHL - Sensorineural hearing loss; MHL- Mixed hearing loss

Although most cases showed a mild hearing loss degree with thresholds below 45 dB HL (12/19 ears = 63.16%), some patients had higher auditory thresholds reaching up to 65 dB HL (5/19 ears = 26.32%), whereas others presented thresholds above 65 dB HL (2/19 ears = 10.52%).

The main types of the tympanometric curve were type-A tympanometric curve (14/19 ears = 73.69%), followed by type-Ad and B curves (3/19 ears each= 15.79% each), and type-C curve, found on both ears of a single patient (2/19 ears= 10.52%).

**Figure 1:**
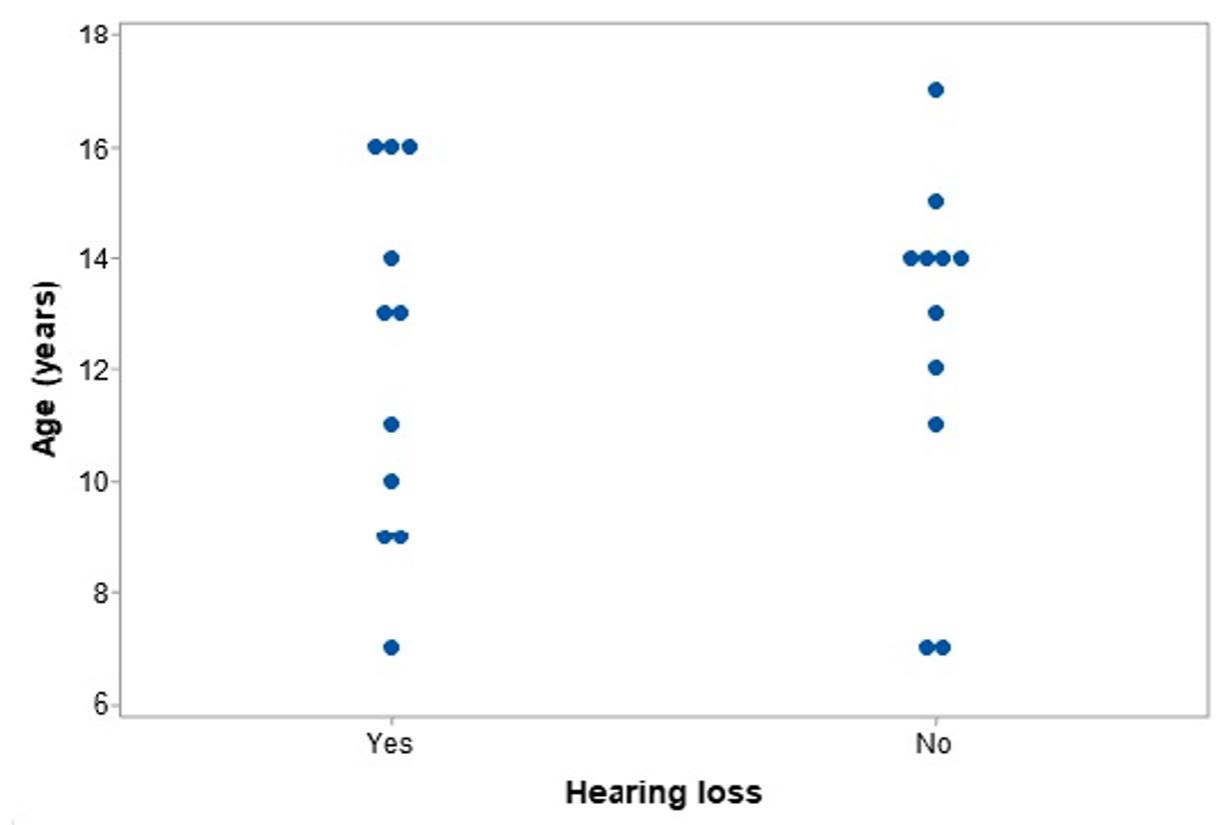
Distribution of patients with or without hearing loss according to chronological age

The analysis of the presence of hearing loss according to the chronological age showed that the two groups (with hearing loss x without hearing loss) did not differ in this respect (t= -0.27; gl= 19; p-value= 0.790), since the data were dispersed, thus not demonstrating any association between these variables (Figure 1).

The comparison of auditory thresholds between the groups revealed significantly higher thresholds in the SG than in the CG (p-value < 0.05) (Table 2).

**Table 2:** Comparison of pure tone audiometry thresholds between both groups

**Frequency**	**Group**	**N**	**Right Ear**		**Left Ear**	
			**Average ± Standard Deviation**	**p-value**	**Average ± Standard Deviation**	**p-value**+
**0.25 kHz**	SG	44	14.77 ± 12.49	0.006*	12.27 ± 9.22	0.003*
	CG	34	6.18 ± 4.52		5.59 ± 2.43	
**0.5 kHz**	SG	44	15.23 ± 12.29	0.001*	12.27 ± 9.22	0.004*
	CG	34	5.00 ± 3.06		5.59 ± 3.49	
**1 kHz**	SG	44	12.50 ± 11.21	0.003*	12.05 ± 12.88	0.004*
	CG	34	4.12 ± 3.18		2.94 ± 3.57	
**2 kHz**	SG	44	11.59 ± 15.30	0.007*	10.23 ± 13.49	0.024*
	CG	34	1.77 ± 3.03		2.95 ± 3.98	
**3 kHz**	SG	44	15.00 ± 18.71	0.009*	12.50 ± 15.41	0.034*
	CG	34	3.23 ± 3.93		4.71 ± 4.83	
**4 kHz**	SG	44	17.50 ± 19.13	0.004*	18.86 ± 21.49	0.012*
	CG	34	4.12 ± 3.63		5.88 ± 5.37	
**6 kHz**	SG	44	21.36 ± 18.78	0.002*	24.55 ± 23.95	0.005^*^
	CG	34	7.06 ± 5.32		8.53 ± 3.43	
**8 kHz**	SG	44	25.23 ± 22.33	0.002*	24.32 ± 19.04	0.000*
	CG	34	7.94 ± 4.70		5.29 ± 4.13	

* p-value statistically significant;

+ p-value obtained through T-test for independent samples

**Caption:** CG- Control Group; SG- Study Group; N- Sampling number; kHz- kilo Hertz

To prevent the functionality analysis of the outer hair cells from being compromised, we measured the TEOAE only in the ears that did not present any type of hearing loss. Thus, 34 ears were assessed in the CG and 25 in the SG. Also, none of these individuals had a record of ear surgery or middle ear alteration, discarded by the presence of tympanometric curve of types B or C without ipsi- or contralateral acoustic reflexes^(14)^.

Initially, we calculated the percentage of responses at each frequency assessed per group. We verified an association between the groups and the presence of TEOAE response, considering the absence of TEOAE as a key differentiator for the WS group (Table 3).

**Table 3:** **-** Percentages of responses in the TEOAE and p-value from the association of variable ‘presence of response’ in relation to SG and CG

**TEOAE**	**Presence of Response**	**SG (n=25)**	**CG (n=34)**	χ^2^	**GL**	**p-value**+
**1 kHz**	Yes	56.00%	88.24%	7.896	1	0.005^*^
	No	44.00%	11.76%			
**1,5 kHz**	Yes	64.00%	100.00%	14.443	1	0.000*
	No	36.00%	0.00%			
**2 kHz**	Yes	52.00%	91.18%	11.662	1	0.001*
	No	48.00%	8.82%			
**3 kHz**	Yes	60.00%	100.00%	16.376	1	0.001*
	No	40.00%	0.00%			
**4 kHz**	Yes	28.00%	64.71%	7.776	1	0.005*
	No	72.00%	35.29%			
***Response***	Yes	64.00%	100.00%	14.443	1	0.000*
	No	36.00%	0.00%			

* statistically significant p-value;

+ p-value obtained through Pearson's chi-square test (χ^2^)

**Caption:** TEOAE- Transient Otoacoustic Emissions; CG- Control Group; SG- Study Group; n- Sampling number; kHz- kilo Hertz

Subsequently, we calculated the descriptive measures considering the signal-to-noise ratio per frequency in each group. Statistically significant differences were found between the groups, with the signal-to-noise ratio with the lowest value in the SG at all analyzed frequencies (Table 4).

**Table 4:** Descriptive analysis and comparison of the signal-to-noise ratio values per frequency analyzed

		**N**	**Average** **(dB SPL)**	**Standard Deviation**	**Minimum**	**Median**	**Maximum**	**T-Value**	**DF**	**p-value**+
**1 kHz**	**SG**	25	3.74	2.51	0.04	3.71	9.44	5.56	48	0.000*
	**CG**	34	9.69	5.52	1.17	7.83	21.53			
**1,5 kHz**	**SG**	25	5.84	4.68	0.10	4.09	16.00	3.96	56	0.000*
	**CG**	34	11.18	5.66	3.62	10.34	24.97			
**2 kHz**	**SG**	25	6.44	5.33	0.13	6.41	15.19	4.11	52	0.000*
	**CG**	34	12.22	5.35	1.27	13.38	21.83			
**3 kHz**	**SG**	25	8.35	5.61	0.18	9.53	16.42	4.74	55	0.000*
	**CG**	34	15.80	6.42	6.07	15.64	26.70			
**4 kHz**	**SG**	25	5.05	4.76	0.39	4.15	20.58	3.42	56	0.001*
	**CG**	34	10.00	6.38	1.92	8.13	25.12			
***Response***	**SG**	25	18.52	4.68	11.35	16.65	27.12	3.14	51	0.003^*^
	**CG**	34	22.40	4.69	13.28	21.70	30.06			

* statistically significant p-value;

+ **p-value obtained through unpaired T-test**

**Caption:** N- Sampling number; dB SPL- decibel Sound Pressure Level; kHz- kilo Hertz; DF- Degrees of freedom

## DISCUSSION

The goal of this study was to assess the audiological profile of individuals with WS and analyze the functionality of outer hair cells of the cochlea in patients without middle ear involvement, a record of ear surgery, or hearing loss. In addition, due to the inclusion of a Control Group, the results obtained from the TEOAE of individuals with WS could be compared with those of individuals with neurotypical development and without audiological involvement.

Our findings point out the relevant prevalence of hearing loss in individuals with WS, thus suggesting that such population has a dysfunction in hair cells, especially in cochlea basal regions, a cochlear involvement that can occur even in patients without any apparent alteration in the basic audiological assessment.

The results from the pure tone audiometry threshold pointed to hearing loss in 50% of the studied population with WS, which corroborates other findings reported in the literature describing a high occurrence of hearing loss in WS cases^(3)^, corresponding from 25% of individuals between 6 and 14 years^(7)^ to 59% of individuals at school age^(5)^. In adult individuals, the literature indicates the presence of hearing loss in more than 38.46% of the assessed cases^(16)^.

We found that most cases involved a mild sensorineural hearing loss emerging especially at high frequencies, from 3 kHz, which corroborates other findings reported in the literature describing the predominance of sensorineural hearing loss^(3-5,8,16,17)^ from mild to moderate degrees^(4-6)^ with major damage at high frequencies^(3,4,6-8,17)^.

Some studies have highlighted that the hearing loss in WS has a similar configuration to the noise-induced hearing loss, probably due to the absence of acoustic reflex, since the physiological mechanism of reflex can protect the auditory system of strong sounds^(4,18)^. In this study, we observed only six ears (13.63%) with auditory lowering at the frequencies of 4 kHz and/or 6 kHz, with recovery at the frequency of 8 kHz, including some presenting reflexes.

It is known that noise-induced hearing loss is related to oxidative stress. Therefore, an increase in the production of reactive oxygen species – ROS can result from uncontrolled mitochondria functions, ischemia, and toxic excitation, causing a production boost of free radicals leaving some regions of the cochlea, especially outer hair cells, vascular striae, and internal hair cells in the cochlea base, which are more vulnerable to lesions^(19)^.

Several studies have assessed the genes involved in the regulation of reactive oxygen species. Even though, the most commonly mentioned genes are not those deleted in the WS, like *GSTM1* ^(20)^, *SOD2*, *PON* ^(21)^, *KCNE1* ^(22)^, *CAT* ^(23)^, *PCDH15*, and *MYH14* gene^(24)^. Thus, the theory of noise-induced hearing loss in WS should be further explored.

It is also worth noting that despite the prominent sensorineural hearing loss in this syndrome, we should not disregard the existence of conductive involvement due to the presence of both type-B and C tympanometric curves and mixed hearing loss, observed in 21.05% of the patients. Other studies reached an even higher percentage in patients with middle ear involvement, corresponding from 23%^(7,16)^ to 47% of the cases^(6)^, in which individuals with WS can be more likely to present middle ear alterations than the general population^(5)^.

Some studies have found that the *elastin* (*ELN*) gene is expressed in many regions of the human body, including the tympanic membrane, auditory tube, and tendons of muscles that sustain the tympanic ossicular chain (as stapedius muscle and eardrum tensor)^(25,26)^. Therefore, the absence of such gene in WS can influence the mobility of the structures in the ossicular-tympanic system that conduct the acoustic stimulus to the internal ear, thus generating a deficiency in the middle ear functionality and consequently conductive hearing loss.

Another highlighted characteristic in the literature is that hearing loss in WS is progressive^(7)^ and has an early onset, starting in adolescence or early adult life^(3,5,8,12,16)^. Although our study ranges a small sampling number of individuals at a limited age group gap (only 10 years), Figure 1 shows a dispersed distribution among the patients both with and without hearing loss. We also found that the patient with the lowest chronological age (seven years) already presents sensorineural hearing loss, which, in addition to confirming the early onset of hearing loss in patients with WS, points out that cochlear involvement can emerge not only during adolescence or early adult life, appearing even in childhood.

Still, concerning the involvement of the cochlear function, our results demonstrated that individuals with WS presented a higher percentage of absent responses and a lower amplitude of TEOAE concerning individuals without the syndrome. Other previous studies had observed the absence or at least reduction of the amplitudes of OAE^(3)^ responses, especially at high frequencies^(4,5)^, even in patients without any evident hearing loss in the assessment of conventional pure tone audiometry^(5,7,12,13,17,27)^.

Some studies also found an attenuation in the TEOAE responses along time^(7,12,17)^. It is worth pointing out that this study observed cochlear involvement in young patients (children and adolescents), which once again confirms that the dysfunction in the cochlea hair cells has an early onset.

It is believed that the deletion of some genes in WS is responsible for the behavioral phenotype presented by these individuals and can explain some of the findings from the audiological assessment.

Many studies consider that the *ELN* gene can be responsible for compromising the cochlear function as well, since it can reduce blood supply in the cochlea via vascular stenosis, resulting in hypoxia and cell death, in addition to causing stiffening of the basilar membrane, deregulating cochlea cell proliferation, and modifying the transduction signal of hair cells^(3)^. Also, a deficiency in the *ELN* gene can lead to an unsynchronized movement of the stereocilia, resulting in delayed activation of the cochlear nerve and hearing loss^(4)^.

In addition to the *ELN*, the *GTF2IRD1* gene was found in the sensorineural tissues of the cochlea (acting as a receptor in the hair cells), spiral ganglion neurons – responsible for triggering the potential of action to conduct the auditory stimulus to the central auditory pathways –, vascular striae, Reissner membrane, among other types of cells in the rat organ of Corti. Thus, such dysfunctions can impair cochlear amplification through disturbances in the ion gradient, thus contributing to the hearing loss found in patients with WS^(10)^.

The *LIMK1* gene has also been associated with the regulation of mobility in the cochlea hair cells, thus its deletion can generate a dysfunction in the mechanisms that regulate the mobility of hair cells and damage cochlear amplification, leading to the early and progressive hearing loss commonly presented by individuals with WS^(9)^.

Additionally, other genes that are often deleted in individuals with WS have been associated with alterations in the organs of the auditory system, like the *FZD9* gene, which has been found in spiral ganglion neurons^(28)^, and the *STX1* gene, observed in the spiral ganglion and synapse of hair cells in the organ of Corti^(29)^.

Accordingly, several studies have highlighted the importance of audiological monitoring in patients with WS, a scope in which OAE analysis is an important resource to be considered in audiological assessment batteries for being able to provide evidence on damages in the function of cochlea hair cells, even before the thresholds reached in the pure tone audiometry became altered^(3-5,7,12,13,17,27)^. Thus, all participants’ families were instructed about the importance of annual hearing monitoring, and the patients with any alteration in the auditory assessment were referred for evaluation and medical conduct.

In this context, together with our findings, it is worth highlighting the importance of introducing public policies aimed at the audiological monitoring for this syndrome, including otorhinolaryngological and audiological assessments that range not only the basics through pure tone and speech audiometry but also acoustic immittance measures and OAE analysis, even in patients without apparent complaints, especially in individuals with difficulties in answering accurately the pure tone audiometry.

Furthermore, the audiological assessment in this study is limited to conventional frequencies. However, since the main involvement of individuals with WS seems to occur in the basal regions of the cochlea, monitoring through high-frequency (above 8 kHz) audiometry could also provide evidence of early alteration in the sensorial hearing organ, and an early diagnosis is important to better guide hearing-related care.

It is also worth emphasizing the difficulty of parents and family members in noticing hearing loss. During the anamneses, none of the parents or caregivers reported considering that the participant had any type of hearing loss. Since hearing losses are generally mild, they can easily go unnoticed in daily life.

Even to a mild degree, hearing loss can have a negative influence on the cognitive skills of attention and memory, compromise learning processes, affect language development, and jeopardize academic performance. Thus, despite being often disregarded, mild hearing loss can compromise the quality of life as a whole, thus requiring proper monitoring by professionals in the area.

## CONCLUSION

Individuals with WS have a high occurrence of hearing loss, especially mild sensorineural and at high frequencies, thus suggesting a dysfunction in hair cells, mainly in the basal regions of the cochlea.

The TEOAE analysis presents some subclinical findings since the alteration in the TEOAE responses was observed in patients without hearing loss, thus representing an important clinical resource to be considered in the routine audiological assessment of these patients.
