# Supplementary material for: Audiological profile and cochlear functionality in Williams syndrome
Source: Codas. 2022 Jan 12;34(3):e20210041. doi: 10.1590/2317-1782/20212021041 (PMC9769433; doi:10.1590/2317-1782/20212021041)
Supplement: Translation

# Translation: O perfil audiológico e a funcionalidade coclear na síndrome de Williams

**Authors:** Liliane Aparecida Fagundes Silva, Rachel Sayuri Honjo Kawahira, Chong Ae Kim, Carla Gentile Matas

**Affiliations:** 1 Departamento de Fisioterapia, Fonoaudiologia e Terapia Ocupacional, Faculdade de Medicina – FM, Universidade de São Paulo – USP – São Paulo (SP), Brasil.; 2 Unidade de Genética, Faculdade de Medicina – FM, Instituto da Criança, Hospital das Clinicas – HC, Universidade de São Paulo – USP – São Paulo (SP), Brasil

**Keywords:** Síndrome de Williams, Perda Auditiva, Cóclea, Audiologia, Audiometria de Tons Puros

## Abstract

**Objetivo:**

avaliar o perfil audiológico e a funcionalidade coclear em indivíduos com SW.

**Método:**

estudo com 39 indivíduos, sendo 22 indivíduos com SW com idade entre 7 e 17 anos, sendo 15 do sexo masculino e 7 do sexo feminino e 17 indivíduos com desenvolvimento típico e normo-ouvintes. Todos os indivíduos foram avaliados por meio da audiometria tonal limiar, medidas de imitância acústica e análise das Emissões Otoacústicas Transientes (EOAT). Foi avaliado o perfil audiológico dos indivíduos com SW, e também foram comparadas as respostas das EOAT entre os indivíduos com SW sem perda auditiva e indivíduos controles.

**Resultados:**

perda auditiva foi observada em 50% dos pacientes, sendo 78,95% neurossensorial e 21,05% mista. Esta perda foi predominantemente de grau leve a moderado, acometendo principalmente as frequências a partir de 3 kHz. Quanto às EOAT, observou-se maior incidência de ausência e de respostas de menor amplitude em indivíduos com SW.

**Conclusão:**

indivíduos com SW apresentam disfunção das células ciliadas, principalmente da região basal da cóclea. Assim, a análise das EOAT é um recurso clínico importante a ser considerada na avaliação audiológica de rotina.

## INTRODUÇÃO

A síndrome de Williams (SW) é uma doença genética rara, decorrente de uma microdeleção na região do braço longo do cromossomo 7 (7q11.23), contendo aproximadamente 28 genes^(1,2)^. Sua incidência é estimada entre 1:7.500 nascidos vivos e possui prevalência igual em ambos os sexos^(1)^. O fenótipo da SW é caracterizado por múltiplas anormalidades físicas, neurológicas e sistêmicas^(2)^.

Quanto ao fenótipo audiológico, a perda auditiva neurossensorial, principalmente nas frequências altas, acima de 3 kHz, tem sido observada em diversos estudos com SW^(3-5)^, sendo que este comprometimento inicia-se no fim da infância ou no começo da vida adulta e tende a ser progressivo^(3,5-7)^.

Afim de compreender este quadro, alguns estudos têm associado a influência de alguns genes frequentemente deletados em indivíduos com SW que podem contribuir para a perda auditiva destes pacientes, entre eles o gene *Elastin* (ELN)^(1)^, o gene *General Transcription Factor* (*GTF2I*) e o gene *Lim Domain Kinase* 1 (*LIMK1*)^(8)^. Diversos estudos têm observado a expressão desses genes em tecidos do sistema auditivo, prejudicando a irrigação sanguínea, o equilíbrio do gradiente iônico e a funcionalidade das estruturas cocleares^(3,9,10)^. Considerando isto, a avaliação audiológica na SW, principalmente no que tange à funcionalidade coclear, é de muita importância para esta população.

Uma forma de mensurar a funcionalidade das células ciliadas externas da cóclea é por meio da captação das Emissões Otoacústicas (EOA) que são respostas geradas pela liberação de energia destas células, que podem ocorrer de forma espontânea ou evocada por meio de algum estímulo acústico. Dentre os estímulos mais utilizados, está o Transiente (EOAT), que é uma estimulação breve por meio de cliques ou *tone burst,* e o Produto de Distorção (EOAPD) onde dois tons puros de diferentes frequências são emitidos simultaneamente e a resposta refletida é um produto de distorção resultante da combinação dos dois tons^(11)^.

Alguns estudos com EOA evidenciaram uma fragilidade colear, sendo que, mesmo indivíduos que apresentaram limiares auditivos dentro da normalidade, podem apresentar alterações cocleares com sintomas sub-clínicos^(4,5,12,13)^. No entanto, estudos relacionados à audição em pacientes com SW ainda são recentes e escassos. Muitas hipóteses foram levantadas desde os primeiros estudos, afim de encontrar respostas que justifiquem o fenótipo comportamental desses indivíduos, no entanto, ainda pouco se sabe sobre a origem da maioria das características auditivas nesta população.

Desta forma, o objetivo do presente estudo é caracterizar o perfil audiológico e avaliar a funcionalidade coclear em indivíduos com SW.

## MÉTODO

Estudo clínico transversal, aprovado pela Comissão de Ética da instituição de pesquisa, sob número 15.825, realizado em indivíduos com SW (Grupo Estudo – GE), atendidos na Unidade de Genética do Instituto da Criança do Hospital das Clínicas da Faculdade de Medicina da Universidade de São Paulo.

Os critérios de inclusão dos indivíduos do GE foram apresentar diagnóstico confirmado da SW pelo *Multiplex Ligation-dependent Probe Amplification* (MLPA) e ter faixa etária entre sete e 17 anos. Quanto aos critérios de exclusão, foram considerados excesso de cera no meato acústico externo ou não realizar todos os procedimentos.

Foi realizada uma análise clínica dos prontuários, por meio da qual 34 pacientes foram selecionados para o estudo; dentre estes, não foi possível obter contato com quatro pacientes, seis pacientes não aceitaram participar, um estava com excesso de cera no meato acústico externo e não retornou para avaliação após remoção, e um não permitiu realizar todos os procedimentos.

Sendo assim, 22 pacientes foram incluídos no estudo, sendo sete do sexo feminino e 15 do sexo masculino, com idade cronológica entre sete e 17 anos (12,36 ± 3,02) e idade mental entre três e 14 anos (6,52 ± 2,28). A avaliação do Quociente de Inteligência (QI), por meio da escala *Wechsler Abreviada de Inteligência* (WASI), mostrou resultados entre 37 e 98 (54,05 ± 13,48).

Para fins de comparação dos resultados, um Grupo Controle (GC) foi composto por uma amostra de conveniência, contando com 17 indivíduos com desenvolvimento típico e com audição normal, sendo sete do sexo feminino e 10 do sexo masculino, com idade cronológica entre sete e 17 anos (11,88 ± 3,12). Esses indivíduos não apresentavam queixas de atraso no desenvolvimento neuropsicomotor cognitivo, de fala ou escolar, que foi verificado por meio de anamnese com os pais ou responsáveis.

Para descartar a presença de perda auditiva ou comprometimento de orelha média nos pacientes do GC, foi considerado como critério de inclusão: presença de curva timpanométrica tipo A, Ad ou Ar e reflexos acústicos presentes nas frequências de 0,5, 1, 2 e 4 kHz, ipsi e contralaterais^(14)^; limiares auditivos obtidos pela audiometria tonal inferiores a 20 dB NA em todas as frequências testadas (0,25, 0,5, 1, 2, 3, 4, 6 e 8 kHz)^(15)^; Limiar de Reconhecimento de Fala até 10 dB NA acima da média tritonal; e Índice de Percentual de Reconhecimento de Fala alcançando, no mínimo, 88% de acertos^(15)^.

Antes de iniciar os procedimentos, os pais leram e assinaram o Termo de Consentimento Livre e Esclarecido e os pacientes assinaram o Termo de Assentimento. Em seguida os pais responderam a uma anamnese audiológica para se obter informações do histórico dos pacientes quanto à fatores que pudessem interferir na avaliação, e foi realizada uma meatoscopia para verificar possível obstrução do meato acústico externo.

Para verificar possível comprometimento de orelha média, as medidas de imitância acústica foram realizadas com o Imitanciômetro marca *Interacustic*, modelo AT235, com sonda de 226 Hz. De acordo com a medida do pico de admitância, a curva timpanométrica foi caracterizada de acordo com os critérios de Jerger^(14)^. Os reflexos acústicos, foram testados manualmente até a intensidade de 110 dB NA, e foram retestados em casos de ausência de reflexos para confirmação dos resultados.

A seguir, as audiometrias tonal e vocal foram realizadas utilizando Audiômetro clínico da marca *Grason-Stadler,* modelo GSI 61, com o paciente dentro de cabina acústica atendendo à norma ANSI S3.1-1991 de quantidade de ruído ambiental. Foram mensurados os limiares tonais por via aérea, nas frequências convencionais (0,25 a 8 kHz), sendo considerados como limiares auditivos normais valores até 20 dB NA^(15)^. Quando qualquer frequência entre 0,5 e 4 kHz apresentava limiares superiores 20 dB NA, foi realizada a audiometria tonal por via óssea para determinar o tipo da perda auditiva^(15)^. Para determinar o grau da perda auditiva foi considerada cada frequência separadamente, sendo: de 25 a 40 dB NA- grau leve; de 45 a 70 dB NA - grau moderado; de 75 a 90 dB NA - grau severo; a partir de 95 dB NA - grau profundo. Para a audiometria vocal, foi utilizada a pesquisa do Limiar de Reconhecimento de Fala (LRF), buscando a menor intensidade em que o indivíduo acertava pelo menos 50% das palavras trissílabas ou polissílabas faladas, e o Índice Percentual de Reconhecimento de Fala (IPRF), onde uma lista de 25 monossílabas era falada na intensidade de 30 dB NA acima do LRF, sendo considerados como normais resultados acima de 88% de acertos^(15)^.

As Emissões Otoacústicas com Estímulo Transiente (EOAT) foram mensuradas apenas nos pacientes que apresentavam limiares auditivos dentro da normalidade e foram realizadas com o equipamento *Smart EP USB Jr* da marca *Intelligent Hearing Systems (IHS 5020)* (Miami-Flórida) com fone 10D, posicionado no meato acústico do paciente com uma oliva de látex. Os parâmetros de coleta contemplaram a aquisição de 1024 estímulos clique com duração de 75 microssegundos, não linear, com taxa de apresentação de 19,30 estímulos por segundo, ganho de 4000 na intensidade de 80 dB NPS. A janela de análise foi mantida aberta em 30 ms, sendo analisadas as frequências de 1, 1,5, 2, 3 e 4 kHz. Para determinar a presença das EOAT, foi determinada estabilidade da sonda ≥70%, reprodutibilidade de resposta ≥50% e foi utilizado o seguinte critério: relação sinal ruído (S/R) maior do que 3 dB NPS para as frequências de 1 e 1,5 kHz e maior do que 6 dB NPS para as demais frequências. Para a *Response*, foi considerada a presença de resposta em pelo menos três bandas de frequência. Em pacientes que apresentaram EOAT ausentes, a sonda foi reposicionada e uma nova mensuração foi realizada, sendo considerada a melhor resposta obtida.

Os dados coletados foram tabulados e foi realizada uma análise estatística descritiva e inferencial utilizando testes paramétricos, uma vez que a amostra seguiu uma distribuição normal. Assim, o teste T não pareado foi utilizado para comparar os grupos controle e estudo e o teste do qui-quadrado de Pearson (χ^2^) foi adotado para verificar a associação entre duas variáveis categóricas, tais como grupos e presença/ausência de resposta.

Para análise estatística dos dados, adotou-se nível de significância ≤ 0,05 (5%). Os valores considerados estatisticamente significantes foram marcados com um asterisco (*).

## RESULTADOS

Embora apenas duas famílias tenham relatado ciência do paciente ter qualquer perda auditiva, observou-se no presente estudo 11 pacientes (19 orelhas) que apresentaram perda auditiva (50% da amostra) (Tabela 1). Dentre as orelhas com perda auditiva, observou-se maior prevalência da perda auditiva neurossensorial (15/19 orelhas = 78,95%), seguida da perda auditiva mista (4/19 orelhas = 21,05%).

**Tabela 1:** Sumarização dos resultados obtidos da curva timpanométrica e da audiometria tonal nos pacientes que apresentaram perda auditiva

**Paciente**	**Orelha Direita**				**Orelha Esquerda**			
	**Tipo de Curva Timpanométrica**	**Perda Auditiva**			**Curva Timpanométrica**	**Perda Auditiva**		
		**Tipo**	**Grau**	**Frequências (kHz)**		**Tipo**	**Grau**	**Frequências (kHz)**
1	A	PANS	Leve	6 e 8	A	PANS	Leve	6 e 8
2	A	PANS	Leve a moderada	6 e 8	A	PANS	Leve	6 e 8
3	A	Limiares normais			A	PANS	Leve	3 a 6
4	C	PANS	Leve	6 e 8	C	PANS	Leve	6 e 8
5	A	PANS	Leve	0,25 e 0,5	A	Limiares normais		
6	A	PANS	Leve	4	A	Limiares normais		
7	Ad	PANS	Leve	0,25 e 6	Ad	PANS	Leve	0,25
8	A	PANS	Moderada	3 a 8	A	PAM	Leve a severa*	Todas
9	B	PAM	Moderada a severa^*^	Todas	A	PANS	Leve a moderada	4 a 8
10	Ad	PANS	Leve	6 e 8	A	PANS	Leve	1, 2, 3, 6 e 8
11	B	PAM	Leve a moderada*	Todas	B	PAM	Leve a moderada*	Todas

* grau da perda auditiva considerando os limiares obtidos na via aérea

**Legenda:** kHz- Kilo Hertz; PANS- Perda auditiva neurossensorial; PAM- Perda auditiva mista

No que tange ao grau da perda auditiva, observou-se que esta foi de grau leve na maioria dos casos, com limiares inferiores à 45 dB NA (12/19 orelhas = 63,16%); no entanto, observou-se alguns pacientes que apresentaram limiares auditivos superiores a este chegando até 65 dB NA (5/19 orelhas = 26,32%) e ainda alguns que apresentaram limiares acima de 65 dB NA (2/19 orelhas = 10,52%).

Quanto ao tipo de curva timpanométrica, observou-se principal ocorrência da curva timpanométrica do tipo A (14/19 orelhas = 73,69%), seguido da curva do tipo Ad e B (3/19 orelhas cada= 15,79% cada) e por último da curva do tipo C observada em apenas um paciente em ambas as orelhas (2/19 orelhas= 10,52%).

Ao analisar a presença de perda auditiva de acordo com a idade cronológica, observou-se que os grupos (com perda auditiva x sem perda auditiva) não diferiram quanto à idade cronológica (t= -0,27; gl= 19; p-valor= 0,790), sendo possível observar que os dados foram dispersos, não demonstrando nenhuma associação entre essas variáveis (Figura 1).

**Figura 1:** Distribuição dos pacientes com ou sem perda auditiva de acordo com a idade cronológica

Ao comparar os limiares auditivos entre ambos os grupos, observou-se limiares significativamente elevados no GE em comparação ao GC (p-valor < 0,05) (Tabela 2).

**Tabela 2:** Comparação dos limiares da audiometria tonal entre os dois grupos

**Frequência**	**Grupo**	**N**	**Orelha Direita**		**Orelha Esquerda**	
			**Média ± Desvio Padrão**	**p-valor**	**Média ± Desvio Padrão**	**p-valor**+
**0,25 kHz**	GE	44	14,77 ± 12,49	0,006*	12,27 ± 9,22	0,003^*^
	GC	34	6,18 ± 4,52		5,59 ± 2,43	
**0,5 kHz**	GE	44	15,23 ± 12,29	0,001*	12,27 ± 9,22	0,004*
	GC	34	5,00 ± 3,06		5,59 ± 3,49	
**1 kHz**	GE	44	12,50 ± 11,21	0,003*	12,05 ± 12,88	0,004*
	GC	34	4,12 ± 3,18		2,94 ± 3,57	
**2 kHz**	GE	44	11,59 ± 15,30	0,007*	10,23 ± 13,49	0,024*
	GC	34	1,77 ± 3,03		2,95 ± 3,98	
**3 kHz**	GE	44	15,00 ± 18,71	0,009*	12,50 ± 15,41	0,034*
	GC	34	3,23 ± 3,93		4,71 ± 4,83	
**4 kHz**	GE	44	17,50 ± 19,13	0,004*	18,86 ± 21,49	0,012*
	GC	34	4,12 ± 3,63		5,88 ± 5,37	
**6 kHz**	GE	44	21,36 ± 18,78	0,002*	24,55 ± 23,95	0,005*
	GC	34	7,06 ± 5,32		8,53 ± 3,43	
**8 kHz**	GE	44	25,23 ± 22,33	0,002*	24,32 ± 19,04	0,000*
	GC	34	7,94 ± 4,70		5,29 ± 4,13	

* p-valor estatisticamente significativo;

+ p-valor obtido pelo teste T para amostras independentes

**Legenda:** GC- Grupo Controle; GE- Grupo Estudo; N- Número amostral; kHz- kilo Hertz

Para não comprometer a análise da funcionalidade das células ciliadas externas, foram mensuradas as EOAT apenas nas orelhas que não apresentaram qualquer tipo de perda auditiva. Assim, avaliou-se 34 orelhas no GC e 25 orelhas no GE. Além disso, nenhum desses indivíduos apresentavam histórico de cirurgia otológica ou alteração de orelha média, que foi descartada pela presença de curva timpanométrica do tipo B ou C com ausência de reflexos acústicos ipsi ou contralaterais^(14)^.

Inicialmente foram calculadas as porcentagens de respostas presentes em cada frequência avaliada, para cada grupo. Foi verificada associação entre os grupos e a presença de resposta das EOAT, sendo que a ausência das EOAT foi um diferencial para o grupo com SW (Tabela 3).

**Tabela 3:** Porcentagens de respostas presentes das EOAT e o p-valor da associação da variável ‘presença de resposta’ em relação aos GE e GC

**EOAT**	**Presença de Resposta**	**GE (n=25)**	**GC (n=34)**	χ^2^	**GL**	**p-valor**+
**1 kHz**	Sim	56,00%	88,24%	7,896	1	0,005*
	Não	44,00%	11,76%			
**1,5 kHz**	Sim	64,00%	100,00%	14,443	1	0,000*
	Não	36,00%	0,00%			
**2 kHz**	Sim	52,00%	91,18%	11,662	1	0,001*
	Não	48,00%	8,82%			
**3 kHz**	Sim	60,00%	100,00%	16,376	1	0,001*
	Não	40,00%	0,00%			
**4 kHz**	Sim	28,00%	64,71%	7,776	1	0,005*
	Não	72,00%	35,29%			
***Response***	Sim	64,00%	100,00%	14,443	1	0,000*
	Não	36,00%	0,00%			

* p-valor estatisticamente significativo;

+ p-valor obtido pelo teste do qui-quadrado de *Pearson* (χ^2^)

**Legenda:** EOAT- Emissões Otoacústicas Transientes; GC- Grupo Controle; GE- Grupo Estudo; n- Número amostral; kHz- kilo Hertz

A seguir, foram calculadas as medidas descritivas considerando a relação S/R para cada frequência em cada grupo. Observou-se diferenças estatisticamente significativas entre os grupos, sendo que a relação S/R foi menor no GE, em todas as frequências analisadas (Tabela 4).

**Tabela 4:** Análise descritiva e comparação dos valores da relação S/R para cada frequência analisada

		**N**	**Média (dB NPS)**	**Desvio Padrão**	**Mínimo**	**Mediana**	**Máximo**	**Valor-T**	**GL**	**p-valor**+
**1 kHz**	**GE**	25	3,74	2,51	0,04	3,71	9,44	5,56	48	0,000*
	**GC**	34	9,69	5,52	1,17	7,83	21,53			
**1,5 kHz**	**GE**	25	5,84	4,68	0,10	4,09	16,00	3,96	56	0,000^*^
	**GC**	34	11,18	5,66	3,62	10,34	24,97			
**2 kHz**	**GE**	25	6,44	5,33	0,13	6,41	15,19	4,11	52	0,000*
	**GC**	34	12,22	5,35	1,27	13,38	21,83			
**3 kHz**	**GE**	25	8,35	5,61	0,18	9,53	16,42	4,74	55	0,000*
	**GC**	34	15,80	6,42	6,07	15,64	26,70			
**4 kHz**	**GE**	25	5,05	4,76	0,39	4,15	20,58	3,42	56	0,001*
	**GC**	34	10,00	6,38	1,92	8,13	25,12			
***Response***	**GE**	25	18,52	4,68	11,35	16,65	27,12	3,14	51	0,003*
	**GC**	34	22,40	4,69	13,28	21,70	30,06			

* p-valor estatisticamente significativo;

+ p-valor obtido pelo teste T não pareado

**Legenda:** N- Número amostral; dB NPS- decibel Nível de Pressão Sonora; kHz- kilo Hertz; GL- Graus de Liberdade

## DISCUSSÃO

O presente estudo teve por objetivo avaliar o perfil audiológico de indivíduos com SW e analisar a funcionalidade das células ciliadas externas da cóclea dentre pacientes que não apresentavam comprometimento de orelha média, histórico de cirurgia otológica ou perda auditiva. Além disso, por contar com um grupo controle, os resultados obtidos nas EOAT de indivíduos com SW puderam ser comparados com o de indivíduos com desenvolvimento neurotípico e sem comprometimento audiológico.

Os achados desde estudo demonstraram importante prevalência de perda auditiva em indivíduos com SW, sugerindo que esta população apresenta uma disfunção das células ciliadas, principalmente das regiões basais da cóclea, sendo que este comprometimento coclear pode estar presente mesmo em pacientes sem nenhuma alteração aparente na avaliação audiológica básica.

Os resultados obtidos por meio da audiometria tonal limiar demonstraram perda auditiva em 50% da população estudada com SW, o que corrobora com outros achado da literatura que relatam uma alta incidência de perda auditiva na SW^(3)^, correspondendo de 25% dos indivíduos entre 6 e 14 anos^(7)^ à 59% dos indivíduos em idade escolar^(5)^; em indivíduos adultos, a literatura demonstra presença de perda auditiva em mais de 38,46% dos casos avaliados^(16)^.

No que tange às características desta perda auditiva, observou-se que esta foi principalmente neurossensorial de grau leve para a maioria dos casos e acometendo principalmente as frequências altas, a partir de 3 kHz, o que corroborou também com os achados da literatura que descreve predominância da perda auditiva neurossensorial^(3-5,8,16,17)^, de grau leve a moderado^(4-6)^ e com principal prejuízo das frequências altas^(3,4,6-8,17)^.

Alguns estudos destacaram que a perda auditiva na SW tem configuração semelhante à da perda auditiva induzida por ruído, e que isto pode ser em decorrência da ausência de reflexo acústico, visto que o mecanismo fisiológico do reflexo pode proteger o sistema auditivo de sons de forte intensidade^(4,18)^. No presente estudo nós observamos apenas 6 orelhas (13,63%) com rebaixamento auditivo nas frequências de 4 kHz e/ou 6 kHz com recuperação na frequência de 8 kHz, sendo que algumas delas apresentaram reflexos presentes.

Sabe-se que a perda auditiva induzida por ruído está relacionada ao estresse oxidativo. Assim, um aumento na produção de espécies reativas de oxigênio (*reactive oxigen species* - ROS) pode ocorrer por descontrole das funções da mitocôndria, isquemia e excitação tóxica, levando a um aumento na produção de radicais livres deixando algumas regiões da cóclea, principalmente as células ciliadas externas, a estria vascular e as células ciliadas internas da base da cóclea, mais vulneráveis a lesões^(19)^.

Diversos estudos, têm avaliado os genes envolvidos na regulação das espécies reativas de oxigênio. Apesar disto, os genes mais citados não são os deletados na SW, tais como, o *GSTM1* ^(20)^ o *SOD2*, o *PON* ^(21)^, o *KCNE1* ^(22)^, o *CAT* ^(23)^, o *PCDH15* e o gene *MYH14* ^(24)^. Desta forma, a teoria da perda auditiva induzida por ruído na SW ainda deve ser melhor estudada.

Cabe ressaltar também que, embora seja notório que a perda auditiva neurossensorial tenha maior incidência nesta síndrome, não se pode ignorar a existência do comprometimento condutivo, tanto pela presença de curva timpanométrica do tipo B e C, quando pela presença de perda auditiva mista, observada em 21,05% dos pacientes. Outros estudos observaram porcentagem até superior de pacientes com comprometimento de orelha média, correspondendo de 23%^(7,16)^ à 47% dos casos^(6)^, sendo que indivíduos com SW podem ter maior chance de apresentar alterações de orelha média do que a população em geral^(5)^.

Alguns estudos observaram que o gene da elastina (*ELN*) é expresso em muitas regiões do corpo humano, incluindo a membrana timpânica, a tuba auditiva e os tendões dos músculos que sustentam a cadeia tímpano ossicular (tais como músculo do estapédio e tensor do tímpano)^(25,26)^. Assim, a ausência deste gene na SW pode impactar na mobilidade das estruturas do sistema tímpano-ossicular que conduzem o estímulo acústico para a orelha interna, gerando uma deficiência na funcionalidade da orelha média, e consequente perda auditiva condutiva.

Outra característica destacada na literatura é que a perda auditiva na SW é progressiva^(7)^ e de surgimento precoce, iniciando na adolescência ou no começo da vida adulta^(3,5,8,12,16)^. O presente estudo contou com um pequeno número amostral e considerou para avaliação indivíduos com um intervalo de faixa etária restrito (apenas 10 anos). Apesar disto, observou-se na Figura 1 uma distribuição dispersa, tanto dentre os pacientes que apresentaram perda auditiva, quanto dentre os pacientes que não a apresentaram. Também, observou-se que o paciente com a menor idade cronológica (sete anos), já apresentou perda auditiva neurossensorial. Este resultado, além de confirmar o aparecimento precoce da perda auditiva em pacientes com SW, destaca que o comprometimento coclear pode surgir não apenas na adolescência ou início da vida adulta, mas ainda na infância.

Ainda no que tange ao comprometimento da função coclear, os resultados do presente estudo demonstraram que indivíduos com SW apresentaram maior porcentagem de respostas ausentes e de menor amplitude das EOAT em comparação aos indivíduos sem a síndrome. Outros estudos publicados anteriormente já haviam observado ausência ou ainda diminuição das amplitudes das respostas das EOA^(3)^, principalmente para as frequências altas^(4,5)^, mesmo em pacientes sem nenhuma perda auditiva evidente pela avaliação da audiometria tonal convencional^(5,7,12,13,17,27)^.

Alguns estudos também observaram atenuação das respostas das EOAT ao longo do tempo^(7,12,17)^. Destaca-se que no presente estudo observou-se comprometimento coclear em pacientes jovens (crianças e adolescentes), o que confirma mais uma vez que a disfunção das células ciliadas da cóclea se inicia precocemente.

Acredita-se que a deleção de alguns genes na SW seja responsável pelo fenótipo comportamental que esses indivíduos apresentam e podem explicar alguns achados encontrados na avaliação audiológica.

Diversos estudos acreditam que o gene da *ELN* também pode ser responsável por comprometer a função coclear, pois é capaz de reduzir a irrigação sanguínea na cóclea por estenose vascular, resultando em hipóxia e morte celular; também, pode enrijecer a membrana basilar, desregular a proliferação celular da cóclea, e ainda modificar a transdução do sinal das células ciliadas^(3)^. Além disso, uma deficiência no gene da *ELN* pode levar à movimentação dessincronizada dos estereocílios, resultando em atraso na ativação do nervo coclear e em perda auditiva^(4)^.

Além da *ELN*, o gene *GTF2IRD1* foi observado nos tecidos neurossensoriais da cóclea (servindo como um receptor das células ciliadas), nos neurônios do gânglio espiral que são responsáveis por desencadear o potencial de ação para conduzir o estímulo auditivo para as vias auditivas centrais, na estria vascular, na membrana de Reissner e em vários outros tipos de células do órgão de Corti de ratos. Sendo assim, estas disfunções podem prejudicar a amplificação coclear por meio de um distúrbio no gradiente iônico, e contribuir para a perda auditiva verificada nos pacientes com SW^(10)^.

Também, o gene *LIMK1* tem sido associado à regulação da mobilidade das células ciliadas da cóclea. Desta forma, a deleção deste gene pode levar a uma disfunção dos mecanismos que regulam a mobilidade das células ciliadas e prejudicar a amplificação coclear, levando à perda auditiva precoce e progressiva comumente observada em indivíduos com SW^(9)^.

Além desses, outros genes que se encontram frequentemente deletados em indivíduos com SW, têm sido associados às alterações dos órgãos do sistema auditivo, tais como o gene *FZD9*, que tem sido observado nos neurônios do gânglio espiral^(28)^, e o gene *STX1*, que tem sido observado no gânglio espiral e na sinapse das células ciliadas do órgão de Corti^(29)^.

Visto isso, diversos estudos destacaram a importância do monitoramento audiológico em pacientes com SW, sendo que a análise das EOA é um importante recurso a ser considerado na bateria de avaliação audiológica, por tratar-se de um método capaz de dar indícios de prejuízos na função das células ciliadas da cóclea, mesmo antes dos limiares obtidos pela audiometria tonal estarem alterados^(3-5,7,12,13,17,27)^. Sendo assim, todas as famílias dos participantes do presente estudo foram orientadas quanto à importância do monitoramento anual da audição, e os pacientes com qualquer alteração na avaliação auditiva foram encaminhados para avaliação e conduta médica.

Considerando isto, em conjunto com os achados do presente estudo, deve-se destacar a importância da inclusão de políticas públicas voltadas ao monitoramento audiológico nesta síndrome, incluindo avaliação otorrinolaringológica, bem como, avaliação audiológica que contemple, além da avaliação básica por meio da audiometria tonal e vocal, e as medidas de imitância acústica, a análise das EOA, mesmo em pacientes sem queixas aparentes, e principalmente em indivíduos que apresentem dificuldades para responder fidedignamente à audiometria tonal.

Além disso, o presente estudo foi limitado à avaliação audiológica nas frequências convencionais. No entanto, como indivíduos com SW parecem ter principal comprometimento das regiões basais da cóclea, o monitoramento por meio da audiometria de altas frequências (acima de 8 kHz) poderiam também dar indícios de alteração precoce do órgão sensorial da audição, e o diagnóstico precoce é importante visando melhor orientação quanto aos cuidados da audição.

Cabe destacar, também, a dificuldade dos pais e familiares em perceber a perda auditiva. Durante a anamnese, nenhum dos pais ou responsáveis relataram achar que o participante da pesquisa teria qualquer tipo de perda auditiva. Como a perda auditiva geralmente é de grau leve, esta pode passar facilmente despercebida na vida diária.

A perda auditiva, mesmo de grau leve, pode influenciar negativamente nas habilidades cognitivas de atenção e memória, comprometer o aprendizado, afetar o desenvolvimento de linguagem e prejudicar o rendimento acadêmico. Desta forma, a perda auditiva de grau leve, embora seja ignorada em muitos casos, pode comprometer a qualidade de vida como um todo, e por esse motivo é necessário que seja acompanhada com a devida importância por profissionais da área.

## CONCLUSÃO

Indivíduos com SW apresentam alta incidência de perda auditiva principalmente neurossensorial de grau leve e nas frequências altas, sugerindo uma disfunção das células ciliadas principalmente das regiões basais da cóclea.

A análise das EOAT demonstrou achados subclínicos, visto que alteração nas respostas das EOAT foram observadas em pacientes sem perda auditiva, e assim é um recurso clínico importante a ser considerado na avaliação audiológica de rotina destes pacientes.
